# The use of social media as an influence on vaccination rates: A systematic review protocol

**DOI:** 10.1371/journal.pone.0334114

**Published:** 2025-12-18

**Authors:** Juliana Iscarlaty Freire de Araújo, Francisco de Assis Moura Batista, Marilia Rute De Souto Medeiros, Bárbara de Oliveira Sena, José Adailton da Silva, Fábia Barbosa de Andrade

**Affiliations:** 1 Department of Health Sciences, Federal University of Rio Grande do Norte, Natal, Rio Grande do Norte, Brazil; 2 Department of Nursing, Federal University of Rio Grande do Norte, Natal, Rio Grande do Norte, Brazil; 3 Department of Public Health, Federal University of Rio Grande do Norte, Natal, Rio Grande do Norte, Brazil; 4 Department of Public Health Nursing, Federal University of Paraíba, João Pessoa, Paraíba, Brazil; Norbert Wiener University, PERU

## Abstract

Vaccine hesitancy has emerged as one of the major challenges to global public health, exacerbated by the phenomenon of the infodemic—a surge of misinformation amplified through social media platforms. During the COVID-19 pandemic, this dynamic significantly undermined public trust in vaccines. In this context, the present study protocol outlines a systematic review aimed at identifying and synthesizing evidence regarding the influence of infodemia on social media networks and its impact on vaccination coverage. The review will be conducted in accordance with the Joanna Briggs Institute (JBI) Manual for Evidence Synthesis and guided by the PRISMA-P (Preferred Reporting Items for Systematic Review and Meta-Analysis Protocols) statement. The protocol has been registered in the PROSPERO database (CRD42024581283). The research question was formulated using the PECO framework, where the population of interest is the general public; the intervention is the use of social media; the comparator is non-use of social media or use of other media channels; and the primary outcome is vaccination rates. Both qualitative and quantitative studies with primary or secondary data, published in English, Portuguese, or Spanish, will be included. Searches will be performed across major databases including PubMed, Scopus, Web of Science, Embase, Medline, and Lilacs, as well as gray literature through Google Scholar and OpenGrey. Two independent reviewers will conduct study screening and data extraction using EndNote and Rayyan software. Methodological quality will be assessed using tools such as ROBINS-I and COREQ. This systematic review is expected to contribute evidence to guide health communication strategies, strengthen immunization campaigns, and inform public policies aimed at combating digital misinformation.

## Introduction

Vaccines are among the most successful public health interventions in history, preventing millions of deaths annually from infectious diseases [1]. However, their collective effectiveness depends on public trust and adherence, factors increasingly shaped by the digital informational ecosystem, particularly on social media [1–3].

Global public health faces the persistent challenge of insufficient vaccination coverage, which may increase the burden of endemic infectious diseases and the risk of resurgence of previously eliminated pathogens. This decline in vaccination rates is a multifactorial phenomenon, influenced by a complex interaction of elements, including structural barriers such as access to health services, distribution logistics, and socioeconomic constraints, as well as the growing anti-vaccine movement [3] associated with vaccine hesitancy (VH).

Recognized by the World Health Organization (WHO) as one of the top ten threats to global health, VH is characterized by the refusal or delay in accepting vaccination, largely grounded in concerns about vaccine efficacy and safety [3–5], and influenced by a range of context-specific determinants.

The relative importance of these factors is dynamic, evolving with sociopolitical events and public health crises. Crucially, in the digital era, these concerns and narratives are predominantly disseminated and amplified through online social media platforms and other communication channels [6–8].

Access to information through the internet, particularly via platforms such as Facebook, Twitter, WhatsApp, and Instagram, has strengthened anti-vaccine movement. Users of these media tend to select content aligned with their beliefs and values, forming echo chambers that disregard divergent information and create polarized groups with similar ideas, thereby sharing and reinforcing an anti-vaccine narrative [1,9].

Social media (SM) platforms have the power to rapidly disseminate both accurate and inaccurate information on issues of global public interest. While they offer an unprecedented opportunity to educate the population and spread evidence-based information, these platforms also serve as vehicles for the propagation of misinformation and conspiracy theories, which, in the context of this study, fuel VH [5,10,11].

False information has undeniably strengthened VH, a phenomenon that became more visible in society, particularly during the COVID-19 pandemic, bolstered by the internet and the spread of fake news [8], resulting in an informational disorder.

Misinformation plays a crucial role in reinforcing VH, a phenomenon that became particularly evident during the COVID-19 pandemic. This situation—termed infodemic by the WHO—refers to the overload of both accurate and false information, which hinders health decision-making [12,13].

There is an evident interrelationship between exposure to misinformation and vaccination outcomes. This can be conceptualized as follows: exposure to incomplete or false information leads to the construction of inadequate and distorted knowledge, which shapes beliefs and negative attitudes, resulting in VH. Collectively, this manifests as low vaccination coverage rates [5,8,14].

In this context, it is crucial to understand the association between social media use and its influence on vaccination perceptions, as well as to develop effective strategies to combat misinformation, fostering clear, accurate, and evidence-based communication [11].

Despite the growing number of studies on VH and misinformation, the literature still presents gaps regarding how social media–driven infodemics directly influence vaccination coverage rates. In light of this, the present study protocol aims to identify and synthesize the available evidence on the impact of exposure to infodemics on social media on vaccination coverage, focusing on intention to vaccinate, recommendation, and adherence.

**Research question**

The objective of this systematic review is to answer the following research question: “What is the association between misinformation about vaccines on social media and vaccination rates?”

## Methodology

This protocol outlines a systematic literature review that will be conducted in accordance with the methodology provided by the Joanna Briggs Institute (JBI) Manual, 2010, and guided by the Preferred Reporting Items for Systematic Reviews and Meta-Analyses Protocols (PRISMA-P) 2020 checklists [15,16].

The inclusion of scientific articles aims to map key concepts, types of evidence, and research gaps, systematically synthesizing existing knowledge on the topic [15,17].

The study will follow these steps: (1) Formulation of the research question, (2) Identification of relevant studies, (3) Study selection, (4) Data extraction and coding, (5) Analysis and interpretation of results.

This review is registered with the PROSPERO International Prospective Register of Systematic Reviews under the code CRD42024581283.

1. Formulation of the Research Question

To guide the research question, we using the PECO strategy (Population – Exposure – Comparison – Outcome). The structure is as follows (Table 1):

**Table 1:** Key Concepts for the Research Question According to DeCS | Natal – RN, 2024.

Concepts	Definitions
General population	The total number of inhabitants in a specific location. It may be classified as absolute or relative population (demographic density) [18]. This includes individuals eligible for vaccination, who may be analyzed in subgroups (e.g., parents of children, health professionals, older adults, adolescents).
Social media	• Individuals connected by family, work, or shared interests. Also includes computer-based communication networks. • Platforms enabling computer-based communication among individuals with common interests, such as family or work-related ties. • Social media: Platforms that enable users to create and publish information accessed via the Internet. These platforms are typically characterized by user-generated content, high interactivity between creators and users, and integration with other sites. • Use is specified by platform (Facebook, Instagram, X/Twitter, TikTok, WhatsApp), content valence (pro-vaccine, neutral, anti-vaccine/misinformation), type of use (passive exposure, active engagement), and intensity (frequency, duration, reach).
Vaccination rates	The extent of immunization services, expressed as the proportion of individuals who have been effectively immunized relative to the population that should have received immunization [19].

Source: Authors, 2024.

**P (Population)** – General population

**E (**Exposure) – Use of social media

**C (Comparison)** – No use of social media or use of other communication methods

**O (Outcome)** – Vaccination rates

Therefore, the research question that will guide this review is: What is the association between misinformation about vaccines on social media and vaccination rates?

### Inclusion and exclusion criteria

This protocol is for a mixed-methods systematic review, allowing for a comprehensive synthesis. Relevant primary and secondary data studies will be included, encompassing observational quantitative designs (cross-sectional, case-control, cohort, and ecological studies), as well as qualitative studies exploring perceptions and experiences, such as phenomenology, ethnography, grounded theory, case studies, and research based on interviews or focus groups that investigate the influence of social media on vaccination coverage.

The following will be excluded: editorials, letters to the editor, conference abstracts, news articles, commentaries, review studies. Dissertations and theses will not be included due to challenges related to standardization and access, as well as the risk of data duplication, since their main findings are often subsequently published in peer-reviewed articles. This decision may introduce residual publication bias, which will be acknowledged and discussed as a study limitation. When necessary, study authors will be contacted to clarify or provide missing but presumed available data.

Studies will be limited to those published in English, Portuguese, and Spanish, and to open-access sources. Following Cochrane recommendations, language restrictions should be transparent and justified [20]. This choice is based on feasibility, as the research team’s language proficiency is concentrated in these three languages, ensuring accurate data extraction and analysis without the risk of translation errors. Appraising and extracting data from studies in languages not mastered by the team would increase the risk of misinterpretation and inconsistency in qualitative or quantitative analyses. Furthermore, most relevant scientific literature, particularly in public health, is available in English [21]. We acknowledge that this approach may introduce linguistic bias, which will be evaluated and discussed in detail in the final manuscript.

### Databases to be searched

Searches will be conducted in the following electronic databases: Medline, PubMed, LILACS via BVS, Web of Science, Scopus, and Embase. For gray literature, searches will be carried out using Google Scholar and Open Grey.

The search process will involve systematically retrieving records from databases without year restrictions, using strategies adapted for each database. Screening will be conducted as recommended by the JBI Manual (2010) [15] in two phases: first, titles and abstracts will be screened for preliminary selection; subsequently, full texts of potentially relevant studies will be assessed for eligibility. This step will be performed independently and blindly by two reviewers, with disagreements resolved by a third reviewer.

#### Search strategy.

The search terms and strategy were developed collaboratively by all members of the research team. Two groups of keywords were used, related to: (a) social media and (b) vaccination. Initially, controlled vocabularies in health sciences were used, including Health Sciences Descriptors (DeCS), Medical Subject Headings (MeSH), and Emtree (Embase), to identify multiple descriptors for each topic and to obtain broader results from the databases. Boolean operators OR and AND were applied.

A preliminary search was conducted to identify additional free-text terms for inclusion in the main search strategy. The final search string was tailored for each database used in this review. This strategy was developed with the support of a professional librarian, an expert in systematic review methodology (Table 2).

**Table 2:** Database Search Strategy. Natal, RN, 2024.

Database	Strategy
**PUBMED**	(“Social Networking”[mh] OR “social network”[tiab] OR “Networking, Online Social”[tiab] OR “Social Media”[mh] OR “Media, Social”[tiab] OR “Social Medium”[tiab] OR Facebook[tiab] OR Twitter[tiab] OR Instagram[tiab] OR tiktok[tiab] OR “Fake News”[tiab]) AND (“Immunization Schedule”[mh] OR Vaccination[mh] OR Vaccin*”[tiab] OR Immuniz*[tiab] OR “Vaccination Coverage”[mh] OR “Vaccination Coverage*”[tiab] OR “Immunization Coverage”[mh] OR “Coverage, Immunization”[tiab] OR “Mass Vaccination”[mh] OR “Vaccination, Mass”[tiab] OR “Mass Immunization”[tiab] OR “Immunization, Mass”[tiab] OR “Anti-Vaccination Movement”[mh] OR “Anti-Vaccination Group*”[tiab] OR “Anti Vaccination Group*”[tiab] OR “Group, Anti-Vaccination”[tiab] OR Antivaccinat*[tiab] OR “Vaccination Refusal”[mh] OR “Refusals, Vaccination”[tiab] OR “Refusal, Vaccination”[tiab] OR “Vaccination Refusal*”[tiab] OR “Vaccine Refusal”[tiab] OR “Movements, Anti-Vaccination”[tiab] OR Antivax[tiab] OR “anti-vaxer”[tiab] OR “Anti-Vaccine Movement”[tiab] OR “Vaccination Refusal”[mh] OR “Refusal, Vaccination”[tiab] OR “refusal of vaccination”[tiab] OR “Vaccination Refusals”[tiab] OR Vaccine Refusal”[tiab] OR Vaccines[mh] OR “vaccine hesitancy”[tiab] OR “Vaccination Hesitancies”[tiab] OR “Vaccination Hesitancy”[mh] OR “Vaccination Delay*”[tiab] OR “Delays, Vaccination”[tiab] OR “Vaccine Hesitancies”[tiab] OR “Delays, Vaccine”[tiab] OR “Vaccination Delay*”[tiab])
**Web of Science**	“Social Networking” OR “social network” OR “Networking, Online Social” OR “Social Media” OR “Media, Social” OR “Social Medium” OR Facebook OR Twitter OR Instagram OR tiktok OR “Fake News”) (Title) and (“Immunization Schedule” OR Vaccination OR Vaccin* OR Immuniz* OR “Vaccination Coverage*” OR “Immunization Coverage” OR “Coverage, Immunization” OR “Mass Vaccination” OR “Vaccination, Mass” OR “Mass Immunization” OR “Immunization, Mass” OR “Anti-Vaccination Movement” OR “Anti-Vaccination Group*” OR “Anti Vaccination Group*” OR “Group, Anti-Vaccination” OR Antivaccinat* OR “Vaccination Refusal” OR “Refusals, Vaccination” OR “Refusal, Vaccination” OR “Vaccination Refusal*” OR “Vaccine Refusal” OR “Movements, Anti-Vaccination” OR Antivax OR “anti-vaxer” OR “Anti-Vaccine Movement” OR “Vaccination Refusal” OR “Refusal, Vaccination” OR “refusal of vaccination” OR “Vaccination Refusals” OR “Vaccine Refusal” OR “vaccine hesitancy” OR “Vaccination Hesitanc*” OR “Vaccination Delay*” OR “Delays, Vaccination” OR “Vaccine Hesitancies” OR “Delays, Vaccine” OR “Vaccination Delay*”) (Topic)
**SCOPUS**	TITLE=(“Social Networking” OR “social network” OR “Networking, Online Social” OR “Social Media” OR “Media, Social” OR “Social Medium” OR Facebook OR Twitter OR Instagram OR tiktok OR “Fake News”) AND TITLE-ABS-KEY=(“Immunization Schedule” OR Vaccination OR Immunization OR “Vaccination Coverage” OR “Immunization Coverage” OR “Coverage, Immunization” OR “Mass Vaccination” OR “Vaccination, Mass” OR “Mass Immunization” OR “Immunization, Mass” OR “Anti-Vaccination Movement” OR “Anti-Vaccination Group” OR “Anti Vaccination Group” OR “Group, Anti-Vaccination” OR “Vaccination Refusal” OR “Refusals, Vaccination” OR “Refusal, Vaccination” OR “Vaccination Refusal” OR “Vaccine Refusal” OR “Movements, Anti-Vaccination” OR Antivax OR “anti-vaxer” OR “Anti-Vaccine Movement” OR “Vaccination Refusal” OR “Refusal, Vaccination” OR “refusal of vaccination” OR “Vaccination Refusals” OR “Vaccine Refusal” OR “vaccine hesitancy” OR “Vaccination Hesitancies” OR “Vaccination Delay” OR “Delays, Vaccination” OR “Vaccine Hesitancies” OR “Delays, Vaccine” OR “Vaccination Delay”)
**EMBASE**	(‘social network’:ti OR ‘social network’/exp OR ‘networking, online social’:ti OR ‘social media’:ti OR ‘media, social’:ti OR ‘social medium’:ti OR ‘facebook’:ti OR ‘twitter’:ti OR ‘instagram’:ti OR ‘tiktok’:ti,kw OR ‘fake news’:ti) AND (‘immunization’/exp OR ‘vaccination’/exp OR ‘vaccin*’:ti,ab,kw OR ‘vaccination coverage’/exp OR ‘coverage, immunization’:ti,ab,kw OR ‘mass immunization’/exp OR ‘mass immunization’:ti,ab,kw OR ‘immunization, mass’:ti,ab,kw OR ‘anti-vaccination movement’/exp OR ‘anti-vaccination group*’:ti OR ‘group, anti-vaccination’:ti,ab,kw OR ‘antivaccinat*’:ti,kw OR ‘vaccination refusal’/exp OR ‘refusals, vaccination’:ti,ab,kw OR ‘vaccination refusal*’:ti,ab,kw OR ‘vaccine refusal’:ti,ab,kw OR ‘refusal of vaccination’:ti,kw OR ‘movements, anti-vaccination’:ti,kw OR ‘anti-vaccine movement’:ti,ab,kw OR ‘refusal, vaccination’:ti,ab,kw OR ‘vaccination refusals’:ti,ab,kw OR ‘vaccine refusal’:ti,ab,kw OR ‘vaccine’/exp OR ‘vaccination hesitancies’:ti,ab,kw OR ‘vaccine hesitancy’/exp OR ‘vaccination delay*’:ti,ab,kw OR ‘vaccine hesitancies’:ti,ab,kw OR ‘delays, vaccine’:ti,ab,kw OR ‘vaccine’/exp)
**LILACS**	(ti:vaccin* OR ti:vacin* OR ti:vacun* OR mj:“Immunization Schedule” OR ti:immuni* OR ti:inmun* OR ti:imuniz* OR mj:”Immunization Programs” OR mj:”vaccination” OR mj:”Vaccination Coverage” OR mh:vaccines OR mh:”Esquemas de Imunização” OR mj:”Cobertura Vacinal” OR mh:”Vacinação em Massa” OR ti:”Vacunación Masiva” OR ti:”Mass Vaccination” OR tw:”Mass Immunization” OR mh:”Movimento contra Vacinação” OR ti:”Grupos contra Vacina” OR ti:”Anti-Vaccination Movement” OR mh:”Recusa de Vacinação” OR mj:”Hesitação Vacinal” OR ti:”Vaccination Refusal” OR tw:”Refusals, Vaccination” OR ti:”Refusal, Vaccination” OR ti:”Vaccine Refusal” OR tw:”Refusal, Vaccination” OR ti:”refusal of vaccination” OR ti:”Vaccine Refusal” OR ti:”vaccine hesitancy”) AND (mj:”Rede social” OR ti:”Uso de Rede Social” OR ti:”Social Networking” OR ti:”social network” OR mh:Mídias Sociais OR ti:”Networking, Online Social” OR ti:”Social Media” OR ti:”Social Media” OR ti:”Media, Social” OR ti:”Social Medium” OR ti:Facebook OR ti:Twitter OR ti:Instagram OR ti:tiktok OR ti:”Fake News”)
**Google Scholar**	(“vaccine refusal” OR vaccine) AND (Facebook OR Twitter OR Instagram OR tiktok OR “Fake News”)
**Open Grey**	(“vaccine refusal” OR vaccine) AND (Facebook OR Twitter OR Instagram OR tiktok OR “Fake News”)

Source: Authors, 2024.

### Study selection from evidence sources

After retrieving records from all databases, the studies will be exported to EndNote reference manager (Clarivate Analytics, PA, USA) for duplicate removal. Then, the records will be imported into Rayyan QCRI® (Qatar Computing Research Institute, Doha, Qatar), a platform used to blind the reviewers through its blinding feature. The inclusion and exclusion of documents will be based on predefined eligibility criteria.

Two reviewers will independently conduct the selection process. Any disagreements will be resolved through discussion and consensus with a third reviewer. A standardized checklist based on the eligibility criteria will be used. In cases of missing data, study authors will be contacted for clarification. Studies that do not meet the inclusion criteria will be excluded.

Before data collection begins, a pilot test will be conducted with all reviewers to minimize bias and ensure a consistent selection process. Each author will screen a sample of articles based on titles and abstracts, using the established inclusion criteria. The team will then discuss any discrepancies to determine whether modifications to the criteria or definitions are necessary. Screening will begin only after achieving an agreement level ≥ 75%, as measured by Fleiss’ Kappa statistics [22].

#### Data extraction and management.

A structured data extraction form will be created using Microsoft Excel to collect the following data from eligible studies: Study characteristics (year of publication, author, journal, title, country); Methodology; Type of study; Study location; Sample characteristics (age range, gender); Use of primary or secondary data; Influence of social media on health decision-making; Reported vaccination coverage; Study results. The form will be pilot-tested before implementation, and adjustments will be made as necessary (Tables 3 and 4).

**Table 3:** Characterization of Selected Articles for Analysis. Natal, RN, 2024.

Article	Title	Reference	Journal and Database

Source: The Authors, 2024.

**Table 4:** Studies Comprising the Sample According to Their Objective, Study Type, and Main Findings. Natal/RN, 2024.

Method	Objective	Sample	Main Findings

Source: The Authors, 2024.

Reviewers will extract data independently. In the event of discrepancies, a third reviewer will be consulted to achieve consensus. Outcome completeness for each study will be documented; studies lacking essential information for analysis will not be automatically excluded. Corresponding authors will be contacted via email to provide missing data or clarifications. Gaps will be recorded, and, when necessary, sensitivity analyses will be conducted to assess the impact of incomplete data [20]. The selection process will be documented in a PRISMA-P flow diagram, following the Preferred Reporting Items for Systematic Reviews and Meta-Analyses Protocols [16].

For data synthesis, a segregated convergent synthesis approach will be adopted, as recommended by the JBI Manual (2020) [23], for mixed-methods systematic reviews. Quantitative findings will be synthesized narratively and independently, while qualitative findings will be grouped using thematic synthesis methodology to provide in-depth interpretation of perceptions and experiences. For correlation of results, a convergent integration approach will be applied, organizing points of convergence, divergence, and complementarity in a matrix to ensure a comprehensive understanding of the studied phenomenon [24].

#### Risk of bias assessment.

Validated tools specific to each study design will be used to evaluate internal methodological quality, rather than completeness of reporting alone, thereby ensuring the quality and transparency of the evidence synthesis. For observational quantitative studies (cross-sectional, cohort, case-control), the ROBINS-I tool (Risk Of Bias In Non-randomized Studies – of Interventions) will be applied. For qualitative studies, the Consolidated Criteria for Reporting Qualitative Research (COREQ) [25,26].

Regarding the exclusion of studies based on language, which may represent a potential source of bias, we opted to limit our review to publications in English, due to the predominance of relevant scientific literature in this language. Additionally, Spanish and Portuguese were included to ensure accuracy in data extraction and analysis, as these are languages in which the reviewers are fluent. As highlighted by Stern and Kleijnen (2020), limited resources and language proficiency are common barriers to the inclusion of non-English studies in systematic reviews. When researchers are not proficient in certain languages, the risk of errors in data extraction and interpretation increases, potentially compromising methodological quality. This reinforces the importance of a careful selection of included studies [26,27].

### Ethics

This systematic review does not require approval from a research ethics committee involving human subjects. The review will contribute to the existing body of evidence concerning the use of social media and its influence on vaccination coverage.

The results of this study will be disseminated through publication in an open-access scientific journal, and, when possible, presented at scientific conferences in the field of public health. In the event of any changes to the protocol after publication, relevant updates, including justifications and dates, will be provided and published accordingly.

## Discussion

The use of social media is widespread on a global scale, expanding beyond mere communication channels and having significant implications for public health. The SARS-CoV-2 pandemic in 2020 highlighted a scenario of informational disorder, marked by the massive spread of false information and conflicting narratives. This phenomenon, which had previously emerged in more discreet forms, reached alarming proportions during this period [10,28–30].

Thus, the findings of this systematic review protocol have the potential to provide a comprehensive perspective on the effects of misinformation disseminated via social media on vaccination coverage. By compiling and analyzing the available empirical evidence, this study may shed light on the mechanisms through which the infodemic—characterized by an overload of information and the spread of misleading content—directly influences individual decisions regarding vaccination [11]. Understanding these mechanisms is crucial for addressing vaccine hesitancy, a phenomenon significantly fueled by digital polarization and the circulation of pseudoscientific discourses in online environments [12, 31, 32]].

Nonetheless, some inherent limitations of this field of study must be acknowledged. The dynamic nature of social media, with frequent changes in algorithms, formats, and platforms, may affect the temporal applicability of the findings. Additionally, accurately measuring the impact of misinformation presents a methodological challenge, as the classification of what constitutes “false content” may vary across studies and be influenced by different cultural, political, or epistemological criteria.

Despite these limitations, this review may play a strategic role in guiding responses to the infodemic. The synthesized data could support more effective health communication strategies, help restore public trust in vaccines, and strengthen adherence to vaccination campaigns. By identifying research gaps, this study may also guide future investigations into misinformation and immunization in digital contexts.

Moreover, by offering systematized evidence on the impacts of the infodemic on vaccination coverage, this review could support the development of evidence-based public policies, promote more effective communication strategies, and strengthen public confidence in immunization. In this sense, the study may contribute directly to improving public health indicators by mitigating the effects of misinformation and expanding the reach of vaccination campaigns, particularly in settings of high social vulnerability.

## Conclusion

Vaccine hesitancy, largely driven by the infodemic on social media, stands as one of the most pressing contemporary challenges in global public health. This systematic review protocol aims to identify and synthesize the scientific evidence that explains how the circulation of false, distorted, or sensationalist information in digital environments influences both individual and collective decisions regarding vaccination.

By understanding the impacts of this informational dynamic on vaccination coverage, this review seeks to significantly contribute to overcoming current barriers to immunization. The systematization of data from different contexts will allow not only the mapping of key themes, authors, and methodological approaches, but also the generation of insights into the factors that modulate the spread of misinformation and its reception by users.

This knowledge may foster the development of more assertive, evidence-based, and culturally sensitive communication strategies, aimed at promoting safer informational environments. In a scenario marked by growing polarization and distrust in science, the findings of this study may be decisive for informing public policies focused on media literacy and strengthening trust in health institutions.

By offering an in-depth understanding of the role of social media in vaccine hesitancy, this review may contribute to the design of more effective and sustainable interventions to combat misinformation.

### Implications for research, practice, or policy

The main contribution of this review is to provide evidence on the impact of digital social media on decision-making regarding vaccination, and to explore whether these decisions may be disseminated via media platforms and influence broader audiences. Furthermore, the results of this review will offer valuable insights on the topic, contributing to academic and scientific debate.

The conclusions drawn may also be instrumental in the formulation and advocacy of new public health policies, particularly those that leverage social media as a strategic tool for promoting vaccination.

### Preliminary timeline

The following preliminary timeline outlines the key stages and estimated completion periods for this systematic review (Table 5):

**Table 5:** Stages of the Systematic Review. Natal/RN, 2024.

*Stage*	*Start*	*Completion*
Pilot Searches to Underpin the Review Protocol and Define the Search Strategy	July 20, 2024	August 10, 2024
Development of the Review Protocol	May 20, 2024	August 10, 2024
Protocol Registration in PROSPERO	August 19, 2024	August 30, 2024
Study Selection	August 31, 2024	September 22, 2024
Data Extraction and Coding	September 23, 2024	Not complete
Analysis and Interpretation of the Results	Not started	Not started

Source: The Authors, 2024.

## Supporting information

S1 File Prisma-P 2015 checklist. Checklist prism protocol, Natal, RN, Brazil, 2024.
(PDF)
