# Supplementary material for: The use of social media as an influence on vaccination rates: A systematic review protocol
Source: PLoS One. 2025 Dec 18;20(12):e0334114. doi: 10.1371/journal.pone.0334114 (PMC12714219; doi:10.1371/journal.pone.0334114)
Supplement: S1 File — Checklist prism protocol, Natal, RN, Brazil, 2024. (PDF) [file pone.0334114.s001.pdf]

PRISMA-P (Preferred Reporting Items for Systematic review and Meta-Analysis Protocols) 2015 checklist: recommended items to address in a systematic review protocol*

Section and topic	Item No	Checklist item
**ADMINISTRATIVE INFORMATION**		
Title:		
Identification	1a	Identify the report as a protocol of a systematic review - The Use of Social Media as an Influence on Vaccination Rates: A Systematic Review Protocol
Update	1b	If the protocol is for an update of a previous systematic review, identify as such - not applicable
Registration	2	If registered, provide the name of the registry (such as PROSPERO) and registration number CRD42024581283
Authors:		
Contact	3a	Provide name, institutional affiliation, e-mail address of all protocol authors; provide physical mailing address of corresponding author<br>Juliana Iscarlaty Freire de Araújo, Department of Health Sciences, Federal University of Rio Grande do Norte, Natal, Rio Grande do Norte, Brazil. ju.iscarlaty@hotmail.com. Av. Sen. Salgado Filho, 1787- Lagoa Nova, Natal - RN, 59064-630<br>Marilia Rute De Souto Medeiros, Department of Health Sciences, Federal University of Rio Grande do Norte, Natal, Rio Grande do Norte, Brazil<br>Francisco de Assis Moura Batista, Department of Health Sciences, Federal University of Rio Grande do Norte, Natal, Rio Grande do Norte, Brazil. assisbaptista13@gmail.com.<br>Bárbara de Oliveira Sena, Department of Nursing, Federal University of Rio Grande do Norte, Natal, Rio Grande do Norte, Brazil. barbara.sena.085@gmail.com.<br>José Adailton da Silva, Department of public health, Federal University of Rio Grande do Norte, Natal, Rio Grande do Norte, Brazil. adailton.silva@ufrn.br.<br>Fábia Barbosa de Andrade, Department of Public Health Nursing, Federal University of Paraíba, João Pessoa, Paraíba. Brazil. fabiabarbosabr@gmail.com.
Contributions	3b	Describe contributions of protocol authors and identify the guarantor of the review<br>Conceptualization: Juliana Iscarlaty Freire de Araújo, Fábia Barbosa de Andrade.<br>Data curation: Juliana Iscarlaty Freire de Araújo, Francisco de Assis Moura Batista.<br>Formal analysis: Juliana Iscarlaty Freire de Araújo.<br>Investigation: Juliana Iscarlaty Freire de Araújo.<br>Methodology: Juliana Iscarlaty Freire de Araújo, Bárbara de Oliveira Sena.<br>Supervision: Fábia Barbosa de Andrade.<br>Validation: José Adailton da Silva, Fábia Barbosa de Andrade.<br>Writing – original draft: Juliana Iscarlaty Freire de Araújo, Marilia Rute De Souto Medeiros, Fábia Barbosa de Andrade.

		Writing – review & editing: Juliana Iscarlaty Freire de Araújo, Francisco de Assis Moura Batista, José Adailton da Silva, Fábia Barbosa de Andrade.
Amendments	4	If the protocol represents an amendment of a previously completed or published protocol, identify as such and list changes; otherwise, state plan for documenting important protocol amendments - not applicable
Support:		
Sources	5a	Indicate sources of financial or other support for the review - not applicable
Sponsor	5b	Provide name for the review funder and/or sponsor - not applicable
Role of sponsor or funder	5c	Describe roles of funder(s), sponsor(s), and/or institution(s), if any, in developing the protocol - not applicable
**INTRODUCTION**		
Rationale	6	Describe the rationale for the review in the context of what is already known<br>The polarization of opinions and the presence of anti-vaccine groups on social media pose significant challenges for public health professionals. In this context, it is crucial to understand how social networks influences perceptions of vaccination and to develop effective strategies to counter misinformation, ensuring clear, accurate, and evidence-based communication.<br>Despite the growing body of research on vaccine hesitancy and misinformation, gaps remain in the literature regarding the direct impact of social media-driven infodemia on vaccination coverage rates. A comprehensive mapping of these influences could provide valuable insights for the development of effective strategies. Given this scenario, the present study aims to identify and synthesize the available evidence in the literature regarding the impact of social networks-driven infodemic on vaccination coverage.
Objectives	7	Provide an explicit statement of the question(s) the review will address with reference to participants, interventions, comparators, and outcomes (PICO)<br>P (Population) - General population,<br>I (Intervention) - Use of social networks,<br>C (Comparison) - Non-use of social networks or use of other forms of communication,<br>O (Outcome) - Vaccination rates.<br>Thus, the research question guiding the review is: What is the relationship between the use of Social networks and vaccination rates?
**METHODS**		
Eligibility criteria	8	Specify the study characteristics (such as PICO, study design, setting, time frame) and report characteristics (such as years considered, language, publication status) to be used as criteria for eligibility for the review<br>We will include all relevant quantitative and qualitative studies based on primary and secondary data that investigate the influence of social networks on vaccination coverage, including descriptive and comparative observational studies (cross-sectional, case-control, intervention, cohort, and ecological).<br>Editorials, letters to the editor, presentations at scientific events, news reports, commentaries, review studies, dissertations, and theses will be excluded. If necessary, contact will be made with the study authors to resolve any uncertainties or obtain clarifications regarding omitted data that is presumed to exist.

		The studies will be limited to those published in English, Portuguese, and Spanish, and that are open access. We acknowledge that this may lead to the exclusion of international studies, and we will discuss this as a limitation in our manuscript.
Information sources	9	Describe all intended information sources (such as electronic databases, contact with study authors, trial registers or other grey literature sources) with planned dates of coverage<br>Searches were conducted in the following electronic databases: Medline, PubMed, Lilacs via BVS, Web of Science, Scopus, and Embase. For gray literature, searches will be carried out in Google Scholar and Open Grey. Searches will be performed using titles and abstracts. Additionally, reference lists from identified articles and any systematic reviews completed on similar topics will be examined in order to ensure that no relevant article is overlooked.
Search strategy	10	Present draft of search strategy to be used for at least one electronic database, including planned limits, such that it could be repeated<br>Web of science - "Social Networking" OR "social network" OR "Networking, Online Social" OR "Social Media" OR "Media, Social" OR "Social Medium" OR Facebook OR Twitter OR Instagram OR tiktok OR "Fake News") (Title) and ("Immunization Schedule" OR Vaccination OR Vaccin* OR Immuniz* OR "Vaccination Coverage*" OR "Immunization Coverage" OR "Coverage, Immunization" OR "Mass Vaccination" OR "Vaccination, Mass" OR "Mass Immunization" OR "Immunization, Mass" OR "Anti-Vaccination Movement" OR "Anti-Vaccination Group*" OR "Anti Vaccination Group*" OR "Group, Anti-Vaccination" OR Antivaccinat* OR "Vaccination Refusal" OR "Refusals, Vaccination" OR "Refusal, Vaccination" OR "Vaccination Refusal*" OR "Vaccine Refusal" OR "Movements, Anti-Vaccination" OR Antivax OR "anti-vaxer" OR "Anti-Vaccine Movement" OR "Vaccination Refusal" OR "Refusal, Vaccination" OR "refusal of vaccination" OR "Vaccination Refusals" OR "Vaccine Refusal" OR "vaccine hesitancy" OR "Vaccination Hesitanc*" OR "Vaccination Delay*" OR "Delays, Vaccination" OR "Vaccine Hesitancies" OR "Delays, Vaccine" OR "Vaccination Delay*") (Topic).
Study records:		
Data management	11a	Describe the mechanism(s) that will be used to manage records and data throughout the review<br>After searching all databases for articles, the study records were exported and imported into the reference manager EndNote (Clarivate Analytics, PA, USA) to remove duplicates. Subsequently, the studies were transferred to the software Rayyan QCRI® (Qatar Computing Research Institute, Doha, Qatar), which was used to maintain the blinding of the research through the blind tool available in the software. In addition, the inclusion and exclusion of documents will be performed based on the established eligibility criteria.
Selection process	11b	State the process that will be used for selecting studies (such as two independent reviewers) through each phase of the review (that is, screening, eligibility and inclusion in meta-analysis)<br>All steps will be carried out independently by two reviewers, and any discrepancies will be resolved through discussion and consensus with a third reviewer. A standard checklist containing the eligibility criteria will be used; in cases of insufficient data, the authors will be contacted for further clarification. Studies that do not meet the inclusion criteria will be excluded.
Data collection process	11c	Describe planned method of extracting data from reports (such as piloting forms, done independently, in duplicate), any processes for obtaining and confirming data from investigators<br>Prior to the commencement of data collection, a pilot test involving all authors will be conducted to reduce bias and ensure a consistent selection process: each author will select a sample of documents based on titles and abstracts, and screening will be performed in accordance with the criteria established for the study.

Data items	12	List and define all variables for which data will be sought (such as PICO items, funding sources), any pre-planned data assumptions and simplifications - Data will be extracted that responds to the PICO strategy that makes up the research question: P (Population) - General population,<br>I (Intervention) - Use of social networks,<br>C (Comparison) - Non-use of social networks or use of other forms of communication,<br>O (Outcome) - Vaccination rates.<br>Thus, the research question guiding the review is: What is the relationship between the use of Social networks and vaccination rates?
Outcomes and prioritization	13	List and define all outcomes for which data will be sought, including prioritization of main and additional outcomes, with rationale<br>The results of this study will be published in an open access scientific journal and, when possible, at conferences in the area of public health, with a view to disseminating information and scientific development on the subject.
Risk of bias in individual studies	14	Describe anticipated methods for assessing risk of bias of individual studies, including whether this will be done at the outcome or study level, or both; state how this information will be used in data synthesis<br>The methodological quality of the included studies will be assessed by two independent reviewers using the Joanna Briggs Institute (JBI) Critical Appraisal Checklist for Analytical Cross-Sectional Studies (2008).<br>Regarding the exclusion of studies based on language, we acknowledge that this may represent a potential source of bias. However, we chose to restrict our review to studies published in English, due to the predominance of relevant literature in this language, as well as studies in Portuguese and Spanish, to ensure accuracy in data extraction and analysis, as these are languages in which the reviewers are fluent. Stern and Kleijnen (2020) emphasize that lack of resources and language proficiency are common barriers to including non-English studies in systematic reviews. When researchers are not fluent in a given language, the risk of data extraction and interpretation errors increases, compromising methodological quality. This reinforces the need for careful selection of included studies.
Data synthesis	15a	Describe criteria under which study data will be quantitatively synthesised - Descriptive presentation.
	15b	If data are appropriate for quantitative synthesis, describe planned summary measures, methods of handling data and methods of combining data from studies, including any planned exploration of consistency (such as I^2, Kendall's τ) - not applicable
	15c	Describe any proposed additional analyses (such as sensitivity or subgroup analyses, meta-regression) - not applicable
	15d	If quantitative synthesis is not appropriate, describe the type of summary planned - not applicable
Meta-bias(es)	16	Specify any planned assessment of meta-bias(es) (such as publication bias across studies, selective reporting within studies)<br>A structured data extraction form will be developed using Microsoft Excel to extract data from eligible studies. The information to be extracted includes: study characteristics (year of publication, author, journal of publication, title, and country); method; type of study; study location; characteristics of the selected sample (age range, sex); whether the study was conducted using primary or secondary sources; the influence of social networks on health decision-making; recorded vaccination coverage; results; conclusions; and the study's limitations along with future research suggestions identified by the authors of the included studies. The form will be pilot-tested prior to use, and modifications will be made as necessary.

Confidence in cumulative evidence	17	Describe how the strength of the body of evidence will be assessed (such as GRADE) - will be used GRADE (Grading of Recommendations Assessment, Development and Evaluation) and Preferred Reporting Items for Systematic Reviews and Meta-Analyses Protocols (PRISMA-P) checklist.

*** It is strongly recommended that this checklist be read in conjunction with the PRISMA-P Explanation and Elaboration (cite when available) for important clarification on the items. Amendments to a review protocol should be tracked and dated.** **The copyright for PRISMA-P (including checklist) is held by the PRISMA-P Group and is distributed under a Creative Commons Attribution Licence 4.0.**

From: Shamseer L, Moher D, Clarke M, Ghersi D, Liberati A, Petticrew M, Shekelle P, Stewart L, PRISMA-P Group. Preferred reporting items for systematic review and meta-analysis protocols (PRISMA-P) 2015: elaboration and explanation. BMJ. 2015 Jan 2;349(jan02 1):g7647.
